# Degradation and de novo formation of nine major glucose degradation products during storage of peritoneal dialysis fluids

**DOI:** 10.1038/s41598-022-08123-1

**Published:** 2022-03-11

**Authors:** Sabrina Gensberger-Reigl, Ingrid Weigel, Joachim Stützer, Andrea Auditore, Tim Nikolaus, Monika Pischetsrieder

**Affiliations:** 1 Food Chemistry, Department of Chemistry and Pharmacy, Friedrich-Alexander-Universität Erlangen-Nürnberg (FAU), Nikolaus-Fiebiger-Straße 10, 91058 Erlangen, Germany; 2 Fresenius Medical Care Deutschland GmbH, Frankfurter Straße 6-8, 66606 St. Wendel, Germany

**Keywords:** Drug safety, Bioanalytical chemistry

## Abstract

Reactive glucose degradation products (GDPs) are formed during heat sterilization of glucose-containing peritoneal dialysis fluids (PDFs) and may induce adverse clinical effects. Long periods of storage and/or transport of PDFs before use may lead to de novo formation or degradation of GDPs. Therefore, the present study quantified the GDP profiles of single- and double-chamber PDFs during storage. Glucosone, 3-deoxyglucosone (3-DG), 3-deoxygalactosone (3-DGal), 3,4-dideoxyglucosone-3-ene (3,4-DGE), glyoxal, methylglyoxal (MGO), acetaldehyde, formaldehyde, and 5-hydroxymethylfurfural (5-HMF) were quantified by two validated UHPLC-DAD methods after derivatization with *o*-phenylenediamine (dicarbonyls) or 2,4-dinitrophenylhydrazine (monocarbonyls). The PDFs were stored at 50 °C for 0, 1, 2, 4, 13, and 26 weeks. The total GDP concentration of single-chamber PDFs did not change considerably during storage (496.6 ± 16.0 µM, 0 weeks; 519.1 ± 13.1 µM, 26 weeks), but individual GDPs were affected differently. 3-DG (− 82.6 µM) and 3-DGal (− 71.3 µM) were degraded, whereas 5-HMF (+ 161.7 µM), glyoxal (+ 32.2 µM), and formaldehyde (+ 12.4 µM) accumulated between 0 and 26 weeks. Acetaldehyde, glucosone, MGO, and 3,4-DGE showed time-dependent formation and degradation. The GDP concentrations in double-chamber fluids were generally lower and differently affected by storage. In conclusion, the changes of GDP concentrations during storage should be considered for the evaluation of clinical effects of PDFs.

## Introduction

Peritoneal dialysis is a renal replacement therapy utilizing peritoneal dialysis fluids (PDFs), which contain an osmotic agent such as glucose, lactate buffer, and electrolytes. For microbial safety, PDFs are usually heat-sterilized, but the process leads to the formation of clinically relevant glucose degradation products (GDPs)^1,2^. During the heat sterilization of PDFs, monocarbonyls like acetaldehyde, formaldehyde, furfural, and 5-hydroxymethylfurfural (5-HMF) as well as α-dicarbonyl compounds such as glucosone, 3-deoxyglucosone (3-DG), 3-deoxygalactosone (3-DGal), 3,4-dideoxyglucosone-3-ene (3,4-DGE), glyoxal, and methylglyoxal (MGO) are formed^1–3^. There is strong evidence that GDPs impair the biocompatibility of PDFs and that the formation of advanced glycation end-products from GDPs, for example, limits the long-term treatment with peritoneal dialysis^4,5^. Moreover, some GDPs can induce inflammatory mechanisms, reduced cellular stress response, or apoptosis, which leads to a loss of cell viability^6–8^. Among the GDPs, 3,4-DGE shows the highest cytotoxicity and is responsible for the most pronounced enzyme inactivation^9,10^.

As an alternative to the widely used single-chamber PDFs, double-chamber-bag products have been developed. Sterilizing the glucose-containing solution separately from lactate at a lower pH of about 3 reduces the GDP load^11,12^. Immediately prior to use, the glucose solution is mixed with a buffer from the second compartment yielding a physiological pH of about 7.4.

It is well established that GDPs are mainly formed during heat sterilization, but their contents in PDFs may change when PDFs are exposed to elevated temperatures during storage and transport. Thus, the GDP composition of PDFs used for the treatment of patients may vary considerably from the contents in the freshly produced solution. To our knowledge, only the behaviors of a few selected GDPs in PDFs have been investigated yet^13,14^. Previously, Zimmeck et al. observed that the concentration of 3-DG decreased during six months of storage at 25, 30, and 40 °C, whereas the concentration of 5-HMF increased^13^. Erixon et al. detected an increasing amount of 3,4-DGE and 5-HMF in a conventional PDF after incubation for 21 days at 25, 40, or 60 °C, respectively, while the concentration of 3-DG decreased during the incubation experiments with the exception of the PDFs stored at 25 °C^14^. Another study monitored the UV absorbance at 228 nm (absorption maximum of 3,4-DGE and 5-HMF) and 284 nm (absorption maximum of 5-HMF) of a heat-sterilized fifty percent glucose solution during storage^15^. All three studies revealed that the changes in GDP concentrations are more pronounced at higher temperatures^13–15^. To date, however, no comprehensive quantitative screening of GDP profiles (including up to ten mono- and dicarbonyls) has been performed in stored PDFs. Additionally, the existing data is not sufficient to compare the behaviors of GDPs in single-chamber and double-chamber-bag PDFs during storage.

A comprehensive analysis of the entire GDP profiles in single- and double-chamber PDFs is, however, of high relevance, because it can be expected that different GDP structures react differently during long-term exposure to storage temperatures. To date, it is not sufficiently clear how the concentrations of glucosone, 3-DGal, glyoxal, MGO, formaldehyde, and acetaldehyde change during storage or transport. Individual GDPs have specific physiological effects^10^ so that their concentration levels in the PDFs that are administered to the patients can be of clinical relevance. Therefore, the present study aimed to investigate storage-induced changes of the major mono- and α-dicarbonyl GDPs in single- and double-chamber PDFs. The present test conditions of 50 °C and 45% relative humidity simulated possible storage or transport conditions during the summer season, in hot climate zones, or during overseas transport in non-tempered cargo containers. The GDPs were quantified in the PDFs by ultrahigh-performance liquid chromatography (UHPLC) coupled with diode array detection (DAD) after derivatizing the six major α-dicarbonyls with *o*-phenylenediamine (OPD) and the three major monocarbonyls with 2,4-dinitrophenylhydrazine (DNPH).

## Results

The present study investigated the influence of storage at 50 °C and 45% relative humidity on the GDP profiles of single- and double-chamber PDFs for up to 26 weeks.

### Profiling of α-dicarbonyl and monocarbonyl GDPs

**Figure 1:**
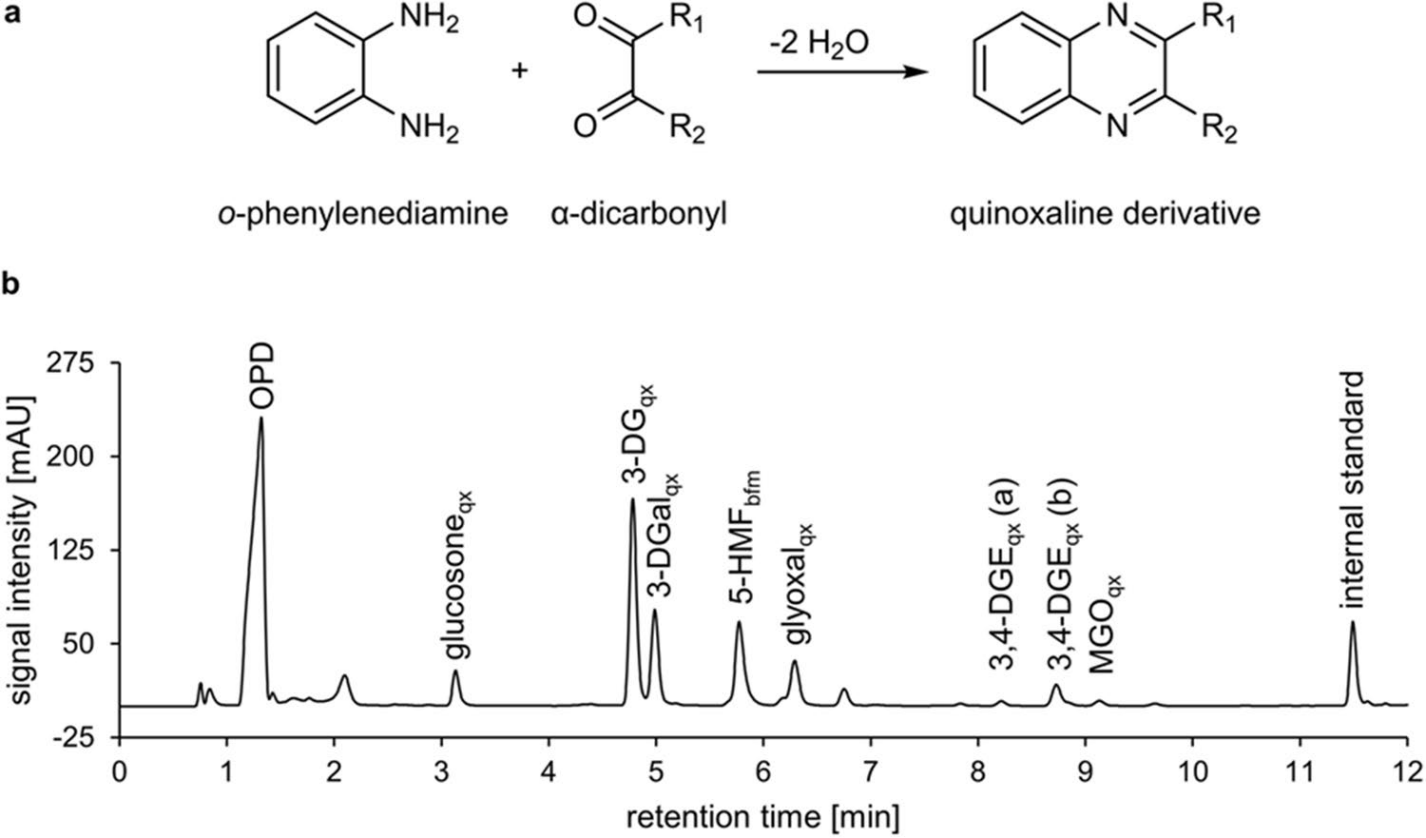
UHPLC-DAD analysis of α-dicarbonyls. (**a**) Derivatizing reaction with OPD to yield corresponding quinoxaline derivatives and (**b**) chromatogram of a typical PDF after derivatization with OPD, recorded at 316 nm. The indices “qx” refer to the quinoxaline derivatives of the α-dicarbonyl GDPs. The index “bfm” refers to (5-(1*H*-benzo[*d*]imidazol-2-yl)furan-2-yl) methanol, which is the benzimidazole derivative of 5-HMF.

Two complementary methods were used for the comprehensive quantitative profiling of GDPs in PDFs. The α-dicarbonyl GDPs were converted with OPD to their respective quinoxaline derivatives (Fig. 1a) using a derivatization method that was previously optimized and validated for the analysis of the six major α-dicarbonyls in PDFs^3^. The monocarbonyl GDP 5-HMF also reacts with OPD yielding a benzimidazole derivative, which gives a signal at 316 nm and can coelute with the quinoxaline derivatives of 3-DGal or glyoxal^16^, thus limiting their accurate quantification. In order to achieve baseline separation of 5-HMF and all major α-dicarbonyls, the pH of the eluent A was set to 3.3 (Fig. 1b).

**Figure 2:**
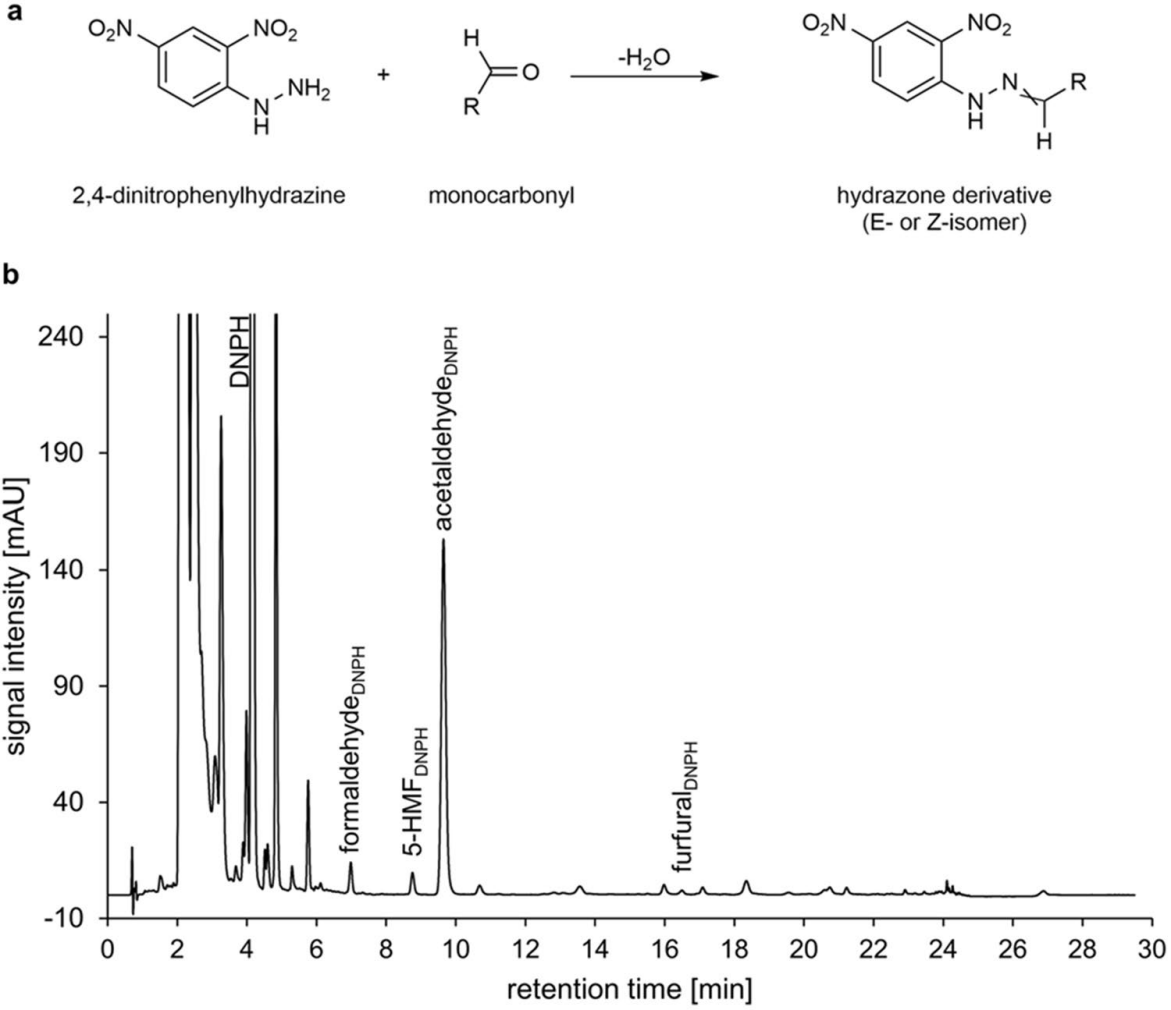
UHPLC-DAD analysis of monocarbonyls. (**a**) Derivatization reaction with DNPH to yield corresponding hydrazones and (**b**) chromatogram of a typical PDF after derivatization with DNPH, recorded at 356 nm. The indices “DNPH” refer to the hydrazone derivatives of the monocarbonyl GDPs. The crossed double bond indicates the formation of E- and Z-isomers.

Since acetaldehyde, furfural, and formaldehyde do not efficiently react with OPD, monocarbonyl GDPs were converted with DNPH into stable hydrazone derivatives (Fig. 2a).

For the analysis of the hydrazone derivatives, a UHPLC-DAD method was established and validated (Fig. 2b). To determine the appropriate time for complete and stable derivatization, the derivatization of the four monocarbonyls 5-HMF, acetaldehyde, furfural, and formaldehyde was monitored between one and twelve and a half hours. The signal for formaldehyde and furfural remained stable over the whole period, whereas the signal of acetaldehyde decreased slightly during prolonged derivatization, but deviated less than 5% within a period of up to eight hours (see Supplementary information, Fig. S-1a). A slight increase of 5-HMF was observed, which was also less than 5% within a period of up to eight hours (Supplementary information, Fig. S-1b).

Thus, a derivatization time between one and eight hours was set. Chromatographic signals for the E- and Z-isomers of the 5-HMF and furfural derivatives could be detected, but only at high concentration levels. At low concentrations, only the signal of the predominant isomer was detectable or quantifiable, respectively. Because the ratio of both isomers was constant, the more intense signal was used for quantification. The calibration curves met the prerequisite of coefficients of determination R^2^ > 0.999 for all calibration models. The relative errors, however, ranged from 74 to 133% and the homogeneity of variances across the concentration range was violated (F-test, P < 0.05). Therefore, we applied weighted linear regression models (Table 1) resulting in relative errors below 5%.

**Table 1:** Calibration models for the quantification of monocarbonyl glucose degradation products in PDFs.

	Concentration range (µM)	Calibration model (weighted)
Furfural	0.2–16.0	y = 0.2175x + 0.0003
5-HMF	0.6–320.0	y = 0.2256x + 0.0000
Formaldehyde	0.8–64.0	y = 0.1747x + 0.0098
Acetaldehyde	1.3–320.0	y = 0.1822x + 0.0000

Recovery and precision were determined at three different concentration levels. In all cases, the recovery did not deviate more than 8.4% from the actual concentration and the variation coefficient was less than 6.3% (Table 2). The results verify that the described procedure is a reliable and precise method to quantify formaldehyde, acetaldehyde, and 5-HMF in glucose-based PDFs.

**Table 2:** Validation parameters of the UHPLC-DAD method for the quantification of monocarbonyls in glucose-containing PDFs after derivatization with DNPH (mean values ± standard deviation of six measurements).

	Added concentration (µM)	Measured concentration ± standard deviation (µM)	Coefficient of variation (%)	Recovery rate (%)
Furfural	1.2	1.3 ± 0.04	3.1	105.7
	4.8	4.6 ± 0.2	4.2	96.3
	9.6	9.2 ± 0.2	1.8	95.9
5-HMF	2.1	2.2 ± 0.07	3.3	102.6
	40.0	39.6 ± 0.1	0.4	99.0
	149.9	148.0 ± 1.9	1.3	98.7
Formaldehyde	1.3	1.2 ± 0.08	6.3	91.6
	10.1	9.7 ± 0.1	1.2	96.0
	18.7	18.2 ± 0.3	1.3	97.5
Acetaldehyde	1.8	1.7 ± 0.09	5.2	93.7
	44.9	43.6 ± 0.5	1.2	97.1
	179.8	175.2 ± 4.6	2.6	97.4

During analysis, a hypsochromic shift at the tail (full width at half maximum) and the apex of the furfural signal was observed in heated PDFs, but neither in standard solutions nor in spiked unheated PDFs (see Supplementary information, Fig. S-2). Therefore, an unknown compound was assumed to coelute with furfural in heat-sterilized glucose-containing PDFs, which may lead to an overestimation of the furfural content. Furfural was the least quantifiable GDP in the PDFs (double-chamber PDFs: ≤ 0.3 µM, single-chamber PDFs: ≤ 1.4 µM; apparent uncorrected concentrations; Table 3). Although furfural shows satisfying validation parameters, we therefore excluded furfural from further analysis. No chromatographic interferences were observed for any of the other GDPs.

**Table 3:** Concentration of four monocarbonyl and six α-dicarbonyl GDPs as well as total GDPs in PDFs in µM after 0 and 26 weeks of storage at 50 °C and 45% relative humidity.

	Week 0		Week 26	
	Single-chamber bag	Double-chamber bag	Single-chamber bag	Double-chamber bag
Formaldehyde	5.0 ± 0.2	n.q.^a^	17.7 ± 0.3	2.5 ± 0.1
Acetaldehyde	129.5 ± 5.9	2.2 ± 0.1	111.6 ± 2.7	1.5 ± 0.2
5-HMF	11.1 ± 0.6	22.5 ± 1.3	172.8 ± 4.6	121.6 ± 3.3
Furfural	≤ 1.4 ± 0.1	≤ 0.3 ± 0.01	n.a.^b^	n.a
Glucosone	25.0 ± 2.7	1.6 ± 0.02	12.0 ± 0.6	6.1 ± 0.01
3-DG	171.1 ± 4.6	28.6 ± 1.1	88.5 ± 4.9	9.4 ± 0.02
3-DGal	127.1 ± 3.9	19.7 ± 0.7	55.9 ± 3.1	n.q
Glyoxal	9.2 ± 0.5	n.d.^c^	41.4 ± 2.5	1.0 ± 0.1
3,4-DGE	10.6 ± 0.4	2.8 ± 0.05	16.1 ± 0.8	n.q
MGO	8.0 ± 0.4	n.d	3.1 ± 0.2	n.d
Total GDP^d^	496.6 ± 16.0	77.6 ± 3.4	519.1 ± 13.1	142.4 ± 3.0

^a^n.q., not quantifiable; ^b^n.a., not assessed because of a coeluting compound; ^c^n.d., not detectable, ^d^excluding furfural.

Mean values ± standard deviation of three different PDF bags are displayed.

### Quantitative profiling of GDPs during storage of single-chamber PDFs

**Figure 3:**
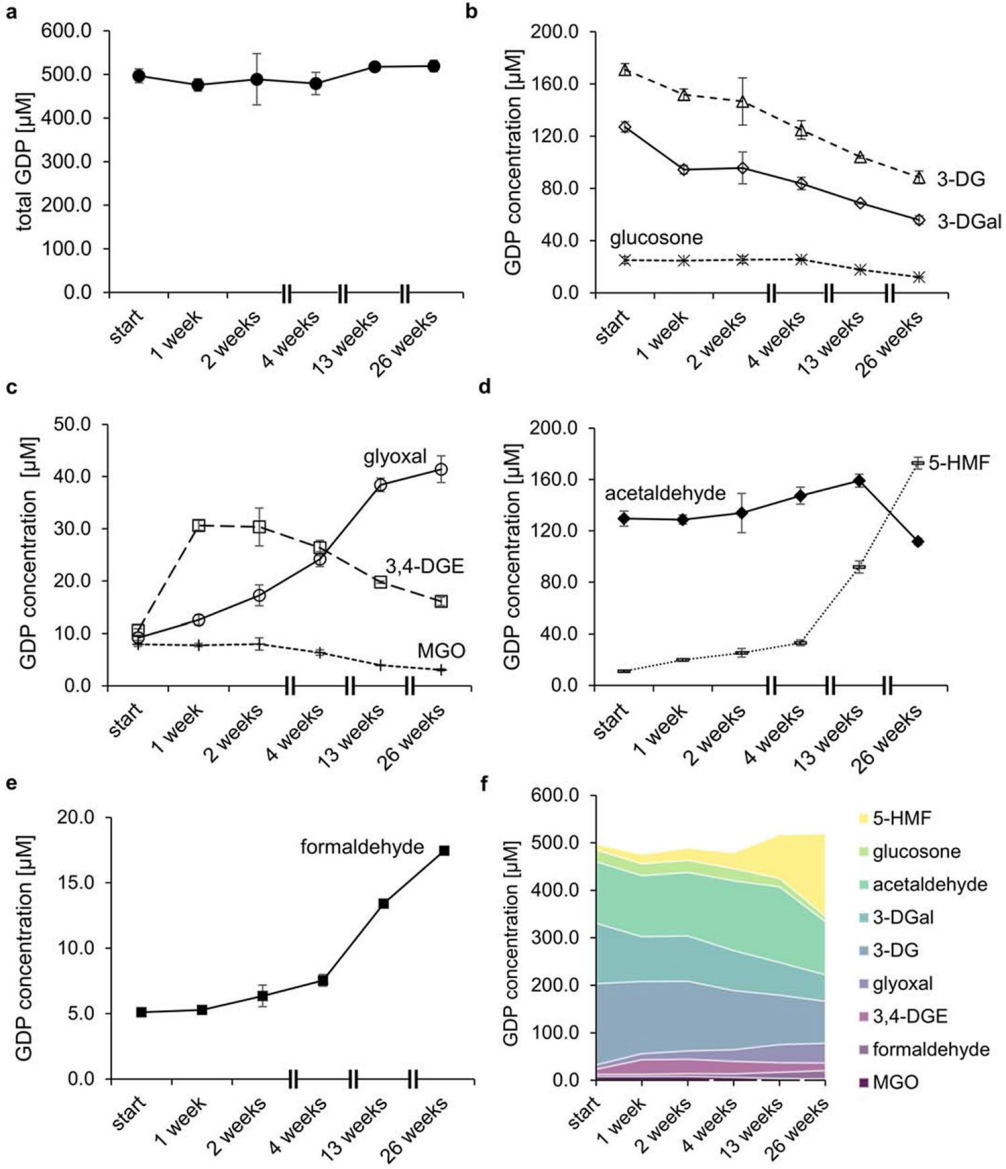
GDP concentration in single-chamber PDFs over storage time. (**a**) Total GDP content (filled circle); (**b**) 3-DG (open triangle), 3-DGal (open diamond), and glucosone (asterisk); (**c**) 3,4-DGE (open square), glyoxal (open circle), and MGO (plus symbol); (**d**) acetaldehyde (filled diamond) and 5-HMF (dashed line); (**e**) formaldehyde (filled square); (**f**) stacked area graph showing all quantified GDPs. Mean values ± standard deviation of three different PDF bags are displayed (except for week 13, for which the mean values ± range of two bags are displayed).

**Figure 4:**
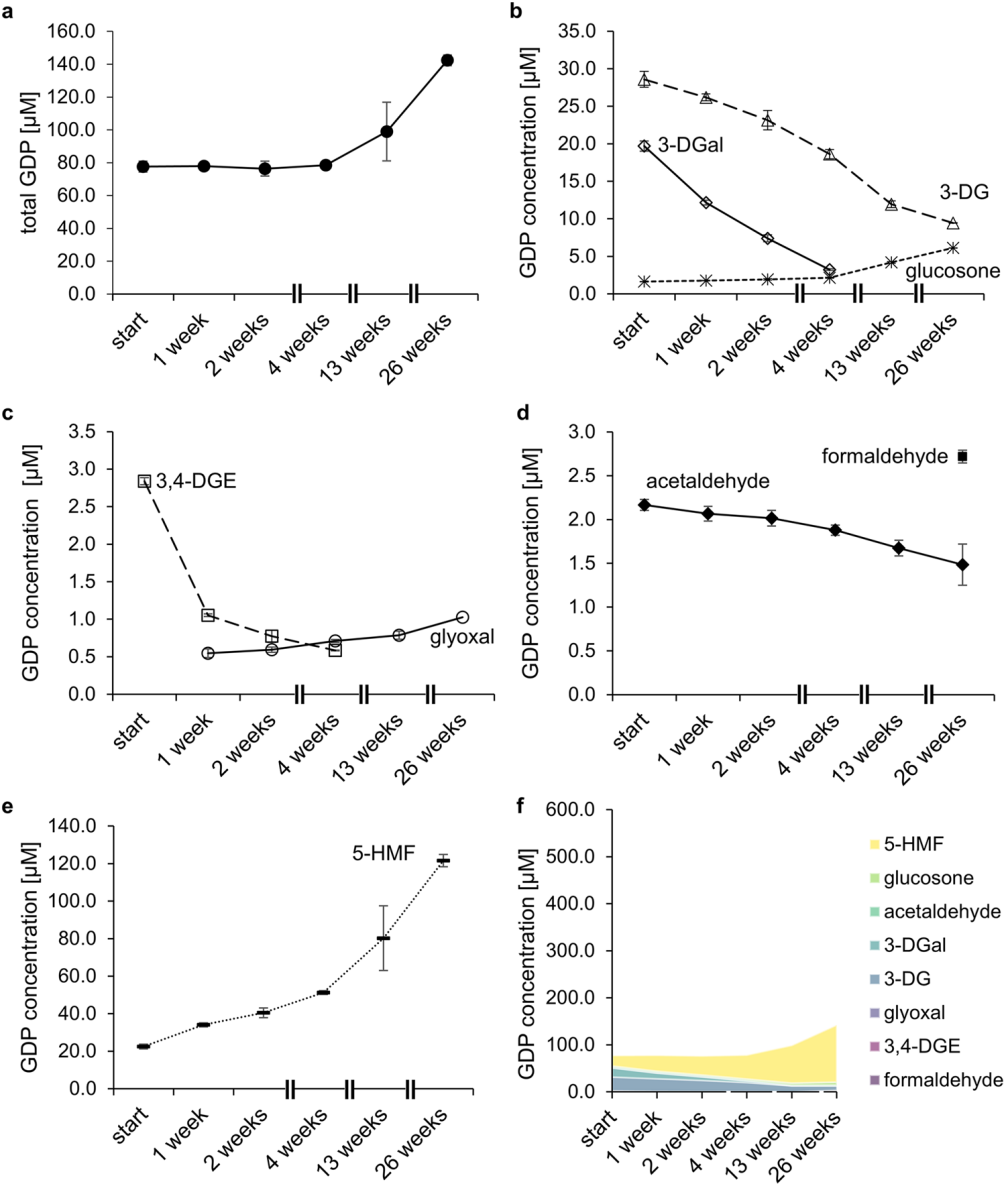
GDP concentration in double-chamber PDFs during storage at 50 °C and 45% humidity. (**a**) Total GDP content (filled circle); (**b**) 3-DG (open triangle), 3-DGal (open diamond), and glucosone (asterisk); (**c**) glyoxal (open circle) and 3,4-DGE (open square); (**d**) acetaldehyde (filled diamond) and formaldehyde (filled square); (**e**) 5-HMF (dashed line), (**f**) stacked area graph showing all quantified GDPs. For better comparison, the scale of the y-axis in (**f**) was adapted to match Fig. 3f. If no data point is displayed, the concentration was below LOQ/LOD. Mean values ± standard deviation in three different PDF bags are displayed.

Changes of the GDP profile in conventional single-chamber PDFs were investigated during storage for up to 26 weeks at 50 °C and 45% relative humidity. The initial total GDP load of 496.6 µM increased slightly to 519.1 µM after 26 weeks of storage (Fig. 3a). The initial contents of 171.1 µM 3-DG and 127.1 µM 3-DGal decreased over time to 88.5 µM 3-DG and 55.9 µM 3-DGal after 26 weeks (Fig. 3b). The concentration of glucosone was stable within four weeks (25.0 µM) and decreased during ongoing storage to 12.0 µM (Fig. 4b).

The MGO content also remained stable within two weeks (8.0 µM) and dropped to 3.1 µM during further incubation (Fig. 3c). The 3,4-DGE concentration increased during the first week from 10.6 to 30.6 µM and decreased afterwards to 16.1 µM within 26 weeks of storage (Fig. 3c). Acetaldehyde was initially present at a concentration of 129.5 µM. Its concentration peaked after 13 weeks (147.4 µM) and decreased later to 111.6 µM (Fig. 3d). The concentration levels of glyoxal, formaldehyde, and 5-HMF rose during the incubation period. At the beginning, the PDFs contained 9.2 µM glyoxal and 5.0 µM formaldehyde. After 26 weeks, 41.4 µM glyoxal and 17.7 µM formaldehyde were present (Fig. 3c,e). The largest increase in concentration was observed for 5-HMF (Fig. 3f). At the beginning of the experiment, 11.1 µM 5-HMF were measured, but the content increased up to 172.8 µM after 26 weeks (Fig. 3d).

### Quantitative profiling of GDPs during storage of double-chamber PDFs

Double-chamber PDFs were stored up to 26 weeks at 50 °C and 45% relative humidity to monitor the GDP contents. Prior to storage, the products contained 77.6 µM GDPs in total. The most abundant GDP was 3-DG (28.6 µM), followed by 5-HMF (22.5 µM), and 3-DGal (19.7 µM). The compounds 3,4-DGE (2.8 µM), acetaldehyde (2.2 µM), and glucosone (1.6 µM) were present at much lower concentrations. Formaldehyde was below the limit of quantification (LOQ; 0.6 µM) and glyoxal below the limit of detection (LOD; 0.2 µM) at week 0. MGO was not detectable in any double-chamber PDF (LOD: 0.2 µM, Table 3).

Up to four weeks of storage, the total GDP load remained constant, followed by an increase to 99.0 µM after 13 weeks and 142.4 µM after 26 weeks (Fig. 4a). As storage progressed, the concentrations of 3-DG, 3-DGal, 3,4-DGE, and acetaldehyde decreased so that neither 3-DGal nor 3,4-DGE was quantifiable at the end of the storage period (Fig. 4b–d). After 26 weeks, only 9.4 µM 3-DG and 1.5 µM acetaldehyde were measured (Fig. 4b,d). During the first four weeks, the glucosone concentration remained almost stable and increased slightly with ongoing storage so that 6.1 µM glucosone was present after 26 weeks (Fig. 4b). Glyoxal, which was not detectable at the beginning, was formed during prolonged storage resulting in 0.5 µM glyoxal after one week and 1.0 µM after 26 weeks (Fig. 4c). Formaldehyde was only quantifiable after 26 weeks (2.5 µM) indicating formation during storage (Fig. 4d, Table 3). The content of 5-HMF increased remarkably during storage (Fig. 4e,f). While 22.5 µM 5-HMF was present at the beginning, its contents increased up to 121.6 µM. At the end of the study, 5-HMF was the major GDP in the double-chamber PDFs (Fig. 4f).

## Discussion

The comprehensive profiling of nine major GDPs in single- and double-chamber PDFs during 26 weeks of storage confirmed the assumption that the GDP concentration and composition in PDFs can differ between fresh products and fluids that patients actually use for dialysis.

PDFs are subject to various storage conditions depending, e.g., on the type of transport (overseas or land transport), the transport routes (long/short), or the ambient temperatures (summer/winter). In particular, temperatures can fluctuate remarkably and may reach almost 60 °C^17^. Since elevated temperatures are expected to have the most severe effects on the GDP composition, the present study assumed worst-case conditions. However, other typical storage conditions, e.g. in dialysis clinics or at home, should be investigated in a next step. Besides, samples drawn from PDFs directly before administration could provide additional information on the actual exposure of patients to GDPs.

The current analysis investigated products ready for dispatch with the consequence that the PDFs had been pre-stored at room temperature for two and five months, respectively. Even though this random selection represented realistic conditions, the pre-storage may be a limitation of the study, because changes of the GDP profile between production and dispatch cannot be excluded.

**Figure 5:**
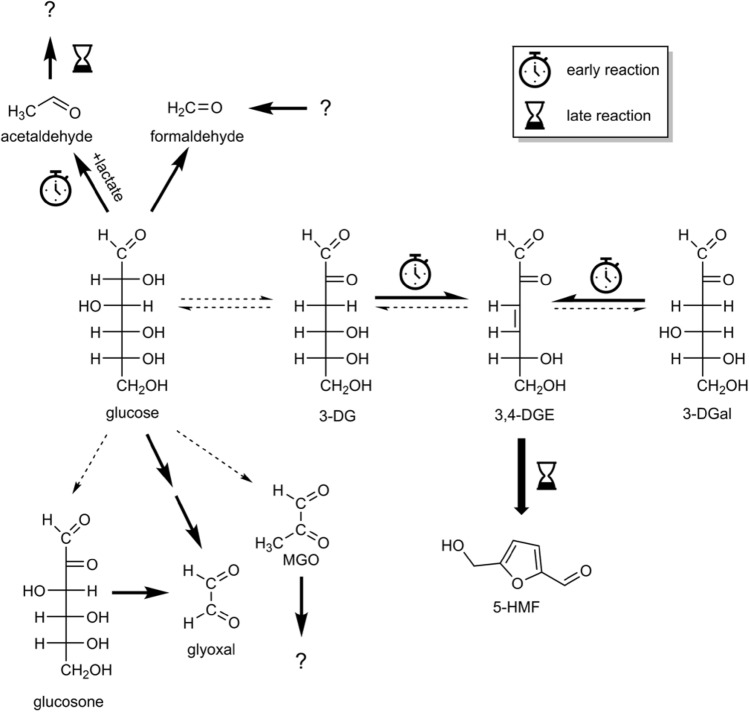
Mechanistic interpretation of changes of the GDP profile in single-chamber PDFs during storage. Reactions leading to changes in the GDP profile are marked with bold arrows; reactions marked with dashed arrows are either not favored during storage or are overcompensated by other reactions and, therefore, do not lead to changes of the GDP profile. Early reactions predominate during short-term storage, whereas late reactions predominate during longer storage periods.

In the present study, the GDP contents in single-chamber PDFs and double-chamber solutions with lower GDP load correspond to the concentration levels previously reported in the literature^2^. In both types of PDF, the concentrations of 3-DG and 3-DGal decreased during prolonged storage. This result is in line with previous studies that also observed a decrease of 3-DG during storage over 21 days at 40 and 60°C^14^, or six months at 25, 30, and 40 °C, respectively^13^. 3-DG and 3-DGal undergo reactions leading, for example, to the formation of 3,4-DGE^9,18,19^ and further to 5-HMF, which seems to be a stable end-product in PDFs^2^ (Fig. 5).

At week 26 of the present storage experiments, we measured additional 161.7 µM 5-HMF and 5.5 µM 3,4-DGE, which corresponds to the degradation of 3-DG (− 82.6 µM) and 3-DGal (− 71.2 µM). Interestingly, the concentration of 3,4-DGE peaked after one week of incubation. Erixon et al. also reported increased 3,4-DGE concentrations, especially at incubation temperatures of 40 °C and 60 °C, during one week of incubation and observed that the 3,4-DGE content decreased again until the end of the 21-day storage period at 60°C^14^. The previous study and our data indicate that the formation of 3,4-DGE from 3-DG and 3-DGal and its degradation to 5-HMF compete with each other at elevated temperatures. Whereas the relatively fast formation dominates at the beginning of storage, it is overcompensated by 3,4-DGE degradation at the end of the storage period (Fig. 5).

The concentrations of glucosone and MGO decreased in single-chamber PDFs over time, but the glucosone content increased slightly in double-chamber solutions. MGO was below LOD in the double-chamber-bag fluids at any time point. Glucosone is formed by the oxidation of glucose^20,21^. Recently, it was shown that traces of redox-active metal ions, such as iron(II), can promote the formation of the oxidized GDP glucosone and, to a lesser extent, also glyoxal and MGO^22^. The concentrations of 3-DG, 3-DGal, and 3,4-DGE, which are formed by non-oxidative mechanisms^2^ were not affected by metal ions^22^. Thus, traces of metal ions from raw materials or production equipment may be responsible for the formation of glucosone during the storage of double-chamber PDFs.

The acetaldehyde content peaked in single-chamber PDFs at week 13, while it decreased in double-chamber bags from the beginning of storage. Acetaldehyde is formed from lactate but is considered as GDP, because its formation is glucose-dependent^1,13^. Single-chamber bags contain lactate and glucose, which mediate the formation of acetaldehyde^13^. In double-chamber bags, glucose and lactate are stored separately so that the reaction is inhibited. Thus, double-chamber bags contain considerably less acetaldehyde compared to single-chamber solutions. Previous studies reported concentrations below LOD or LOQ in double-chamber PDFs, which ranged from 2 to 18 µM, respectively^1,23,24^. The present method is more sensitive, so that acetaldehyde contents between 1.5 and 2.2 µM could be quantified in the PDFs. These results indicate that very minor concentrations of acetaldehyde may be formed from glucose either independently from lactate, during/after mixing the solutions of both compartments, or directly from lactate.

The concentrations of glyoxal and formaldehyde increased over the storage period in both types of PDFs, which may result from the degradation of long-chain GDPs such as glucosone. Glyoxal can be formed from glucosone via retro-aldol cleavage^21^. Alternatively, Yaylayan and Keyhani proposed a mechanism based on the loss of two water molecules from glucose and subsequent retro-aldol reaction^25^. In addition, glyoxal can be formed via oxidation of glycolaldehyde after retro-aldol cleavage of glucose^20^. The reaction mechanism leading to the formation of formaldehyde has yet to be elucidated.

The contents of 3-DG and 3-DGal decreased in both PDF types during 26 weeks of storage. However, 5-HMF, glyoxal, and formaldehyde were formed during the incubation time in both PDF systems. The acetaldehyde content decreased in double-chamber PDFs, while the concentration peaked in single-chamber fluids after 13 weeks. The concentration of 3,4-DGE reached its maximum in single-chamber PDFs after one week of incubation and decreased afterwards. After 26 weeks of storage, however, the 3,4-DGE concentration was still slightly higher than in week 0. In double-chamber bags, a continuous decline of 3,4-DGE was observed. The GDP profiles of single- and double-chamber bags underwent different changes. The PD solutions in both systems vary in pH as well as in glucose, buffer, and electrolyte concentrations. These factors influence the formation and degradation of the GDPs during heat sterilization and subsequent storage. At the beginning, and even at the end of the study, double-chamber PDFs contained less GDPs than single-chamber solutions (Figs. 3f, 4f).

Since it is well established that the individual GDPs feature distinct bioactivities, it is important to consider not only the total GDP content, but also the concentrations of the single GDPs in PDFs. For instance, 3,4-DGE has been reported to have the highest cytotoxicity among the GDPs and to reduce enzyme activity in vitro^9,10^. In single-chamber bags, which contain about threefold higher concentrations of 3,4-DGE compared to double-chamber bags, storage and, in particular, short-term storage may increase its concentration, whereas the storage of double-chamber bag fluids has a beneficial influence on 3,4-DGE concentrations. The biological relevance of 5-HMF is still not clear^26^ and depends highly on the applied concentration. Adverse effects have been described after high doses of 5-HMF in the millimolar range, which reflect or even exceed dietary exposure^27–29^. These effects are mainly linked to the 5-HMF metabolite 5-sulfoxymethylfurfural^29,30^. In contrast, Zhao et al. observed antioxidative properties and antiproliferative activity of 5-HMF against cancer cell lines in vitro^31^. In 2011, the German Federal Institute for Risk Assessment classified 5-HMF as not harmful to health^32^. In PDFs, 5-HMF is present in much lower concentrations and administered peritoneally. In the context of peritoneal dialysis, 5-HMF had no effect on the viability of human peritoneal mesothelial cells^33^ or L-929 fibroblasts^11,33^ in vitro. Morgan et al. reported that 5-HMF did not attenuate the re-mesothelialization of human peritoneal mesothelial cells at concentrations present in PDFs^34^. Although 5-HMF is the most abundant GDP at the endpoint of the present study, it causes less adverse effects in peritoneal dialysis than other compounds such as 3,4-DGE, which are present in lower concentrations.

In conclusion, the described storage effects should be taken into consideration when the clinical relevance of GDPs in PDFs is evaluated.

## Methods

### Chemicals and reagents

All chemicals and reagents were of at least analytical grade and purchased from Sigma-Aldrich (Steinheim, Germany) unless indicated otherwise. Liquid chromatography/mass spectrometry-grade solvents (Carl Roth, Karlsruhe, Germany) and formic acid (Acros, Geel, Belgium) were used in all experiments. 3-DG (purity > 95%) was obtained from Chemos (Regenstauf, Germany). Glucosone^35^, 3-DGal^36^, and 3,4-DGE^19^ were synthesized as reported previously and stock solutions were prepared: glucosone 10 mM, 3-DGal 2.2 mM, and 3,4-DGE 4.9 mM. The concentrations of the stock solutions were determined as described by Mittelmaier et al.^19,35^.

### PDFs and storage conditions

The commercial single-chamber PDFs contained 4.25% (w/w) glucose, sodium d-lactate (3.9 g/L), calcium chloride dihydrate (0.26 g/L), sodium chloride (5.8 g/L), and magnesium chloride hexahydrate (0.10 g/L). The ready-to-use double-chamber PDFs contained 4.25% glucose (w/w), sodium d-lactate (3.9 g/L), calcium chloride dihydrate (0.26 g/L), sodium chloride (5.6 g/L), and magnesium chloride hexahydrate (0.10 g/L). Because the glucose solution and the buffer compartment of double-chamber systems must be mixed prior to sampling, the samples were always drawn from originally sealed bags. Directly after production, the PDFs were kept at room temperature before the present storage experiments started five months (single-chamber PDFs) or, respectively, two months (double-chamber PDFs) after production. For the storage experiments, the PDFs were kept at 50 °C and 45% relative humidity. Samples were drawn after 0, 1, 2, 4, 13, and 26 weeks from three new bags each, except for the single-chamber PDF at week 13, which was only available in duplicate. The study design followed the European Medicines Agency ICH Topic Q 1 A guideline using accelerated testing conditions plus 10 °C to cover extreme temperature scenarios^37^.

### UHPLC-DAD instrument

An Ultimate 3000RS system (degasser, binary pump, autosampler, column oven, and DAD; Thermo Fisher Scientific, Dreieich, Germany) was used with a Waters ACQUITY UPLC® Phenyl column (100 × 2.1 mm, 1.7 μm particle size; Waters, Eschborn, Germany) equipped with a corresponding guard column. System control, data acquisition, and processing were performed by Chromeleon 6.8 software.

### Quantitative profiling of α-dicarbonyl GDPs

α-Dicarbonyl GDPs were quantified as previously reported with minor modifications^3^. Briefly, α-dicarbonyl compounds were converted into their corresponding quinoxaline derivatives by derivatization with OPD. For this purpose, 80 µL of the sample was mixed with 10 µL of derivatizing reagent (4% OPD in 1 M 2-[4-(2-hydroxyethyl)piperazine-1-yl]ethanesulfonic (HEPES) buffer, pH 7) and 10 µL of internal standard (2,3-dimethylquinoxaline, 50 µg/mL in water). The samples were incubated in the dark between 2 and 16 h. Afterwards, the solutions were analyzed by UHPLC-DAD using ammonium formate buffer (5 mM, pH 3.3, eluent A) and methanol (eluent B) at 55 °C with a flow rate of 0.4 mL/min. The eluent composition was [time (minutes)/percent B]: − 3.0/10, 0.0/10, 8.5/25, 10/50, 10.1/80, and 12.0/80. DAD spectra for peak verification were recorded from 190 to 490 nm. 3,4-DGE was quantified at a wavelength of 335 nm, all other analytes at 316 nm by external calibration. The UHPLC-DAD analysis was performed in duplicates.

### Quantitative profiling of monocarbonyl GDPs

For the profiling of monocarbonyl GDPs, all samples were reacted with DNPH according to Tauer et al.^24^ with some modifications. DNPH (10 mg) was dissolved in 4 mL of acetonitrile and 100 µL of 60% sulfuric acid. Aliquots of 500 µL of each PDF were mixed with 200 µL of the DNPH solution, filtered into amber glass vials, and allowed to stand light-protected at room temperature for one (minimum) to 8 h (maximum). The analytes were eluted at 50 °C with a flow rate of 0.4 mL/min using ammonium formate buffer (5 mM, pH 4.5, eluent A) and acetonitrile (eluent B). The eluent composition was [time (minutes)/percent B]: 0.0/20, 1.0/20, 2.5/31, 12.0/31, 13.0/37, 18.0/37, 22.0/55, 22.2/90, 25.0/90, 25.1/20, 30.0/20. The injection volume was 10 µL. DAD spectra for peak verification were recorded from 190 to 600 nm. Except for furfural, no discrepancies between the expected and the recorded UV/Vis spectra were observed in any of the standard solutions or PDFs. Thus, it can be assumed that recovery experiments for 5-HMF, formaldehyde, and acetaldehyde are not negatively influenced in heated PDFs. The analysis of monocarbonyl compounds in PDFs was performed in duplicates.

To test whether the derivatization procedure yielded stable hydrazone derivatives in lactate-buffered PDFs, a commercial single-chamber PDF was treated as described above. UHPLC-DAD analysis was performed after incubation at room temperature for different periods of time up to 12.5 h. Each sample was analyzed in triplicate and the mean values of the peak areas were plotted against the derivatization time.

Formaldehyde and acetaldehyde were quantified at 356 nm and furfural and 5-HMF at 390 nm by external calibration. Since the derivatization reagent contained traces of formaldehyde and acetaldehyde, solvent blanks were analyzed in the same way and the peak areas of formaldehyde and acetaldehyde in the samples were corrected for these background signals. A ten-point calibration curve from 0.6 to 320.0 µM was recorded for 5-HMF and a nine-point calibration curve from 1.3 to 320.0 µM for acetaldehyde. Seven-point calibration curves were obtained for formaldehyde and furfural ranging from 0.8 to 64.0 µM (formaldehyde) and 0.2 to 16.0 µM (furfural). Each calibration level was analyzed in duplicate. The linearity of the calibration curves was evaluated by linear regression analysis with a minimally acceptable coefficient of determination (R^2^) of 0.990, a relative error of < 5%, and homogeneity of variances across the concentration range (F-test, P < 0.05).

To determine recovery rates, an unheated PDF matrix containing 4.25% glucose, lactate buffer, and electrolytes was spiked with furfural (1.2, 4.8, or 9.6 µM), 5-HMF (2.1, 40.0, or 149.9 µM), formaldehyde (1.3, 10.1, or 18.7 µM), or acetaldehyde (1.8, 44.9, or 179.8 µM). Spiked and unspiked PDFs were analyzed as described above. The mean recovery of six experiments for each concentration level was determined and expressed as (analyte concentration − analyte concentration of the unspiked PDF)/added concentration × 100%. Precision was expressed as standard deviation and coefficient of variations.

The LOD and LOQ were determined using the calibration method according to German technical standard DIN 32645:2008-11^38^ and were as follows (LOD/LOQ): formaldehyde 0.1/0.6 µM, acetaldehyde 0.05/0.2 µM, 5-HMF 0.02/0.1 µM, furfural 0.04/0.2 µM.

### Ethical approval

This article does not contain any studies with human participants or animals performed by any of the authors.

## Supplementary Information

Supplementary Information.

## Data Availability

Data is available upon request.
