# Supplementary material for: Degradation and de novo formation of nine major glucose degradation products during storage of peritoneal dialysis fluids
Source: Sci Rep. 2022 Mar 11;12:4268. doi: 10.1038/s41598-022-08123-1 (PMC8917136; doi:10.1038/s41598-022-08123-1)
Supplement: Supplementary file 1 — Supplementary Information. [file 41598_2022_8123_MOESM1_ESM.pdf]

Supplementary Information

Degradation and *de novo* formation of nine major glucose degradation products during storage of peritoneal dialysis fluids

*Sabrina Gensberger-Reigl[1], Ingrid Weigel[1], Joachim Stützer[1], Andrea Auditore[1], Tim Nikolaus[2], Monika Pischetsrieder[1]**

[1] Food Chemistry, Department of Chemistry and Pharmacy, Friedrich-Alexander-Universität Erlangen-Nürnberg (FAU), Nikolaus-Fiebiger-Straße10, 91058 Erlangen, Germany.

[2] Fresenius Medical Care Deutschland GmbH, Frankfurter Straße 6-8, 66606 St. Wendel, Germany

*Correspondence to: monika.pischetsrieder@fau.de

Contents

Figure S-1 Effect of the derivatization time

Figure S-2 UV/Vis spectra of the DNPH derivative of furfural

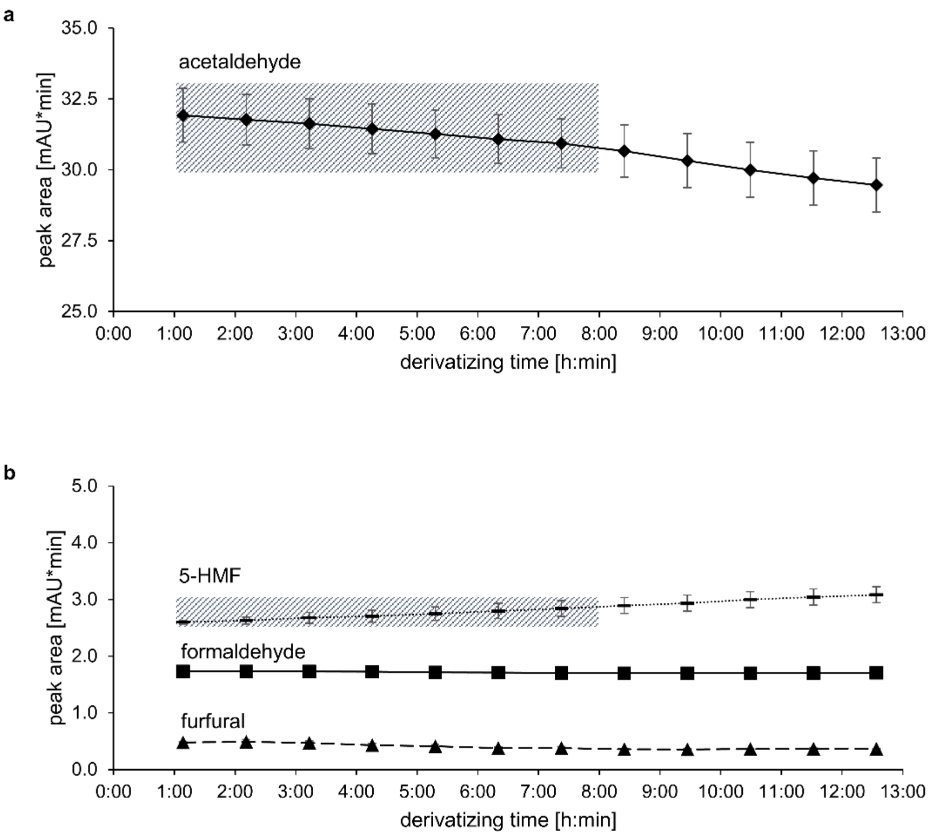

Figure S-1 Effect of the derivatization time on the peak area of (**a**) acetaldehyde (♦) and (**b**) 5-hydroxymethylfurfural (5-HMF, ─), formaldehyde (■), and furfural (▲) in a single-chamber peritoneal dialysis fluid. The grey boxes show the median value from one to eight hours ± 5%.

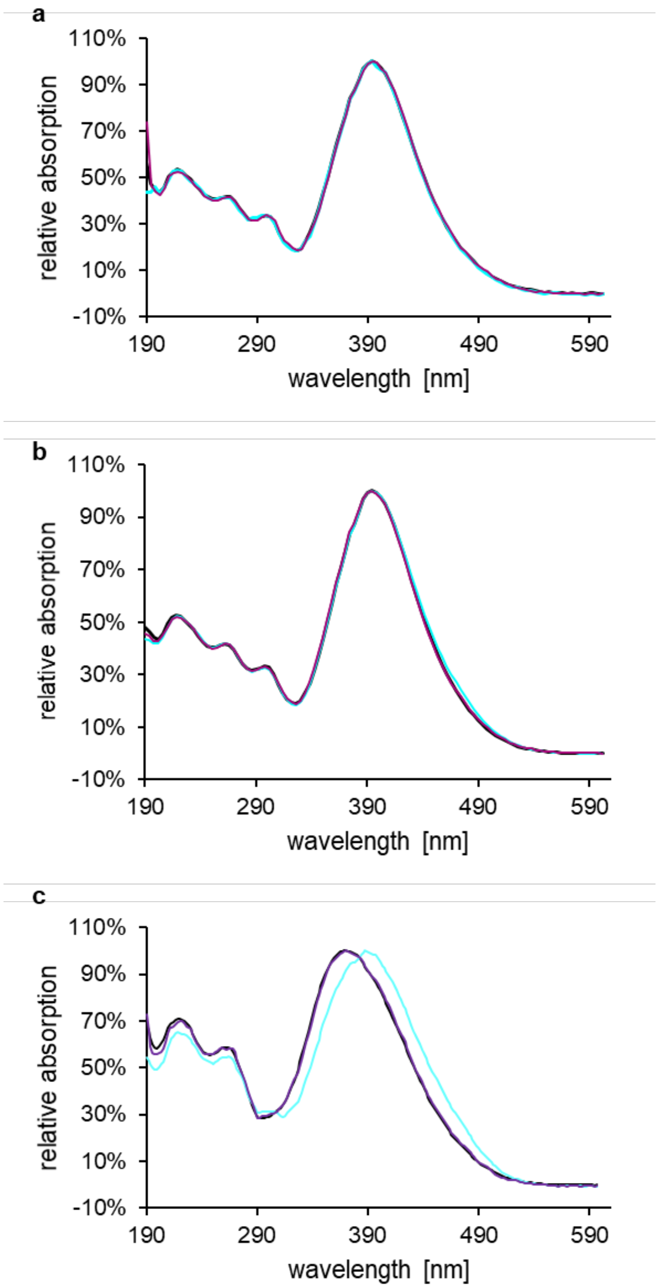

Figure S-2 UV/Vis spectra of the DNPH derivative of furfural in (**a**) solvent, (**b**) unheated PD matrix containing 4.25% glucose, and (**c**) a heat-sterilized single-chamber peritoneal dialysis fluid. Spectra at apex (black) and 50% at full height of the front (turquoise) and tail (purple) are displayed. A hypsochromic shift of the UV/Vis spectra could be observed at 50% at full height at the tail and the apex of the furfural signal in commercial single-chamber peritoneal dialysis fluids.
